# Exploring Exercise as Airway Clearance in Cystic Fibrosis: A Qualitative Study From the ExACT‐CF Feasibility Trial

**DOI:** 10.1002/ppul.71470

**Published:** 2026-01-26

**Authors:** Emily Taylor, Dia Soilemezi, Don S. Urquhart, Steve Cunningham, Steff Lewis, Aileen Rae Neilson, Hannah Ensor, Ioannis Vogiatizis, Lorna Allen, Zoe L. Saynor, the ExACT‐CF study group, Don S. Urquhart, Emily Taylor, Steve Cunningham, Steff Lewis, Aileen Rae Neilson, Dia Soilemezi, Hannah Ensor, Ioannis Vogiatizis, Lorna Allen, Debbie Miller, Donna Bowen, Ellyse Kilarski, Ellen Lacey, Gary Connett, Julian Legg, Hazel Evans, Thomas Daniels, Mark Allenby, Mary Carroll, Robert Gray, Carlos Echevarria, Lisa Morrison, Gordon McGregor, Mary Abkir, Clare Saunders, Zoe L. Saynor

**Affiliations:** ^1^ Department of Child Life and Health University of Edinburgh Edinburgh UK; ^2^ Department of Paediatric Respiratory and Sleep Medicine Royal Hospital for Children and Young People Edinburgh UK; ^3^ Department of Psychology, Faculty of Science and Health University of Portsmouth Portsmouth UK; ^4^ Centre for Inflammation Research University of Edinburgh Edinburgh UK; ^5^ Usher Institute, Edinburgh Clinical Trials Unit, NINE Edinburgh Bioquarter University of Edinburgh Edinburgh France; ^6^ Department of Sport, Exercise and Rehabilitation, Faculty of Health and Life Sciences Northumbria University Newcastle upon Tyne UK; ^7^ Cystic Fibrosis Trust, One Aldgate Second Floor London UK; ^8^ School of Health Sciences, Faculty of Environmental and Life Sciences University of Southampton Southampton UK; ^9^ Wessex Cystic Fibrosis Unit University Hospitals Southampton NHS Foundation Trust Southampton UK; ^10^ National Institute for Health and Care Research, Southampton Biomedical Research Centre University Hospital Southampton NHS Foundation Trust Southampton UK; ^11^ Adult Cystic Fibrosis Unit Western General Hospital Edinburgh UK; ^12^ NIHR Southampton Clinical Research Facility University Hospitals Southampton NHS Foundation Trust Southampton UK; ^13^ Department of Paediatric Respiratory Medicine University Hospital Southampton NHS Foundation Trust Southampton UK; ^14^ Adult Cystic Fibrosis Unit, Royal Victoria Infirmary Newcastle‐upon‐Tyne UK; ^15^ West of Scotland Adult CF Unit Queen Elizabeth University Hospital Glasgow UK; ^16^ Royal Brompton & Harefield Hospitals, part of Guys and St Thomas’ NHS Foundation Trust London UK; ^17^ National Heart and Lung Institute Imperial College London London UK; ^18^ European Cystic Fibrosis Society Lung Clearance Index Core Facility London UK

**Keywords:** chest physiotherapy, cystic fibrosis, physical activity, qualitative, semi‐structured interviews

## Abstract

**Question:**

Cystic fibrosis (CF) affects > 11,300 people in the UK and is characterized by thick, sticky mucus in the lungs, leading to recurrent infections, inflammation, and progressive respiratory decline. Chest physiotherapy remains a cornerstone of airway clearance; however, many people with CF (pwCF) find it burdensome and time‐consuming. Exercise has been proposed as a potentially effective and more acceptable alternative. The ExACT‐CF feasibility trial evaluated the use of exercise as an airway clearance technique in pwCF on modulator therapy.

**Methods:**

This nested qualitative study explored the experiences of participants and healthcare professionals involved in the ExACT‐CF trial, alongside broader perspectives on airway clearance, to inform future research and clinical practice. Purposively sampled semi‐structured interviews were conducted with 32 individuals: ten pwCF, five parents, twelve healthcare professionals, four decliners, and one participant who withdrew. Interviews were audio‐recorded, transcribed verbatim, and analyzed thematically using “The Framework Method.”

**Results:**

Two primary themes emerged. Theme 1 reflected experiences and lessons learned from the trial, including reported barriers and facilitators around recruitment, randomization, and daily implementation. Despite some minor difficulties, trial participation, processes, and the intervention itself were broadly acceptable. Theme 2 highlighted a preference for using ExACT alone or interchangeably with chest physiotherapy. Participants emphasized the need for robust clinical evidence to guide use.

**Answer:**

This study highlights the acceptability of both the ExACT‐CF trial processes and the use of this exercise‐based airway clearance technique. Findings support progression to a definitive trial and further exzploration of patient‐centered, flexible approaches to airway clearance in CF.

**Trial Registration:**

NCT02429180.

## 1 Introduction

Cystic fibrosis (CF) is a life‐long, life limiting condition affecting over 11,300 people in the UK [1]. Airway clearance techniques (ACTs), such as chest physiotherapy combined with mucoactive and/or mucolytic agents, remain essential components of CF care, aiming to mobilize and remove mucus, reduce infection risk, and limit airway inflammation. For over 60 years, daily chest physiotherapy has been a cornerstone of CF management [2], but many people with CF (pwCF) find it time‐consuming [3] and burdensome [4], leading to poor adherence [5, 6]. The CF community has expressed a strong desire to reduce treatment burden [4] and there is increasing interest in exploring whether exercise could serve as a more acceptable and equally effective form of airway clearance [7, 8, 9], as reflected in the top research priorities identified by the James Lind Alliance [9].

The introduction of highly effective modulator therapy (HEMT) has transformed the management of CF for those who have access, significantly reducing pulmonary exacerbations [10], improving life expectancy [11, 12] and, for some, altering ACT requirements. HEMTs enhance secretion hydration and mucociliary clearance [13], and some pwCF report a notable reduction in sputum production [14, 15]. While some individuals have been able to reduce or stop some ACTs, this is not the case for everyone, and ACTs are still considered time‐consuming [16, 17]. As such, the CF community strongly supports clinical evaluations of the safety and impact of simplifying treatment for those on HEMT [18], with the aim of maintaining clinical stability while reducing treatment burden [19].

Enabling more flexible, personalized ACT prescriptions [17, 20] is a key priority for modern CF care. Exercise is increasingly considered a promising option to either supplement or substitute traditional ACTs [21]. While current guidance recommends aerobic and resistance exercise as adjunct therapies to chest physiotherapy [20], many pwCF are independently modifying their regimens based on personal preferences, with some using exercise in combination with other ACTs, other replacing chest physiotherapy altogether [8, 22]. Early studies suggest that certain types of exercise, with forced expiratory maneuvers, may enhance mucociliary clearance [21], but robust evidence is lacking [21, 23].

To address this gap, we developed and tested the feasibility of using the “Exercise as an Airway Clearance Technique” (ExACT) intervention, to evaluate whether exercise could serve as a viable alternative to chest physiotherapy during stable clinical periods. Developed through a Delphi consensus process [24] involving key UK stakeholders and shaped by robust Patient and Public Involvement and Engagement (PPIE), ExACT [25] is a self‐administered, structured aerobic exercise‐based intervention incorporating assessment breaths, huffing and coughing techniques, informed by a recent systematic review [23]. The intervention was evaluated in a pilot randomized controlled trial (RCT) [26], with this paper reporting findings from the embedded qualitative study. We explored the acceptability and reported impact of ExACT, alongside the feasibility of trial procedures, by capturing the experiences of pwCF, caregivers, and healthcare professionals. Specifically, we sought views on the content, delivery, and practicality of the intervention, to inform refinement ahead of a potential definitive trial [27].

## 2 Methods

### 2.1 Study Design and Context

This paper presents findings from the nested qualitative component, designed to capture stakeholder perspectives and explore the feasibility and acceptability of the intervention and trial procedures. ExACT‐CF was a two‐arm feasibility RCT conducted at two UK sites, incorporating mixed‐methods processes which included feasibility, safety and exploratory clinical outcomes, an embedded qualitative study (described in this manuscript) as well as health economic evaluations [25]. Participants were randomized either to the ExACT intervention (stopping their usual ACTs while maintaining mucolytics), or to continue with Usual Care (chest physiotherapy, mucoactive agents, and/or exercise). Trial design and primary outcomes are reported elsewhere [26].

This qualitative sub‐study was underpinned by a phenomenological approach, recognizing that individuals’ experiences are shaped by their unique social and environmental contexts, particularly in the setting of living with CF and participating in a trial [28]. Our aim was to explore and describe the essential meaning of these experiences through a structured yet open inquiry.

### 2.2 Ethics Approval

The study was approved by the Yorkshire and Humber – Sheffield NHS Research Ethics Committee (#22/YH/0223) and Health Research Authority, pre‐registered on ClinicalTrials.gov (NCT05482048), and adopted by the NIHR Applied Research Collaboration Wessex and UK CF Trust Clinical Trials Accelerator Platform. Written informed consent (assent for minors) was obtained from all participants. All names used are pseudonyms.

### 2.3 Participants

Semi‐structured interviews were conducted with purposively sampled participants, including pwCF and parents of children/adolescents from both trial arms. We also included individuals who declined or withdrew from the trial, as well as healthcare professionals (physiotherapists, research nurses, and doctors) involved in trial delivery. Sampling aimed for maximum variation in age, gender, ethnicity, site, trial allocation, and role, guided by a recruitment matrix. Including parents/guardians was essential due to their central role in CF management and the emotional and practical burden they often report [29].

### 2.4 Data Collection

All participants received a detailed information sheet. Verbal consent was obtained prior to interviews, with assent provided by children and adolescents. Interviews were conducted remotely (by telephone or videoconference) at a time convenient for participants, using a semi‐structured guide informed by the literature and interdisciplinary team input (see S2; Online Supplementary Material). Interviews took place between March and October 2023 and were led by three female researchers (ET, DS, ZLS) with backgrounds in physiotherapy, psychology, clinical exercise physiology, and clinical research. Basic sociodemographic data (age, gender, ethnicity) were collected. Interviews were audio‐recorded and transcribed verbatim, either internally or by a certified transcription service under a confidentiality agreement.

### 2.5 Data Analysis

Data were analyzed thematically using “The Framework Method” [30], allowing the integration of both *a priori* themes, and inductive themes emerging from participant narratives. A spreadsheet was used to generate a matrix and the interview data were “charted” into the matrix as a way to systematically map and interpret the data. The initial framework (matrix) was guided by the study's interview guide and expanded to include more codes as new topics were mentioned in the interviews. The framework captured pre‐trial opinions around chest physiotherapy/usual care and exercise, before going on to explore the experiences of recruitment, randomization, trial procedures, and the ExACT intervention, as well as overall trial experience. This framework was used to analyze interview data from participants, as well as interview data from caregivers and health professionals. Two researchers (ET, DS) developed the coding framework in Microsoft Excel, systematically identifying patterns and differences across transcripts.

Initial coding was conducted independently by ET, who reviewed all transcripts and coded data on a preliminary framework matrix. This was iteratively refined through joint coding of selected transcripts and ongoing discussion between the two analysts to ensure consistency. New codes were added inductively as the analysis progressed, enabling flexible categorization of emerging topics. Thematic development was informed by recurring concepts and grounded in an interpretivist stance, recognizing the subjectivity of lived experiences. Regular team meetings supported reflexivity, consensus‐building, and transparency throughout the analysis process.

Relevant sections of The Guidelines on Comprehensive Standards for Reporting Qualitative Research [31] in collecting, analyzing, and reporting information to ensure the transparency and credibility of the research process were followed.

## 3 Results

A total of 32 semi‐structured interviews were conducted with pwCF in the ExACT arm (*n* = 5), Usual Care arm (*n* = 5), parents/caregivers (*n* = 5), decliners/dropouts (*n* = 5), and trial staff (*n* = 12). Most interviews were online; two were by telephone. Interviews lasted an average of 23 min (range: 5–65 min) and were completed within 4 weeks of trial conclusion. Participant demographics are detailed in Table 1. Two overarching themes were identified and are outlined below, with corresponding subthemes summarized in Tables 2 and 3. Additional illustrative quotes for higher order Themes 1 and 2 are provided in the Online Supplementary Material (Tables S1–S2).

**TABLE 1:** Participant demographics.

	Site*	Role/Allocation	Sex (M/F)	Age (years)	Ethnicity
1	A	Participant ‐ ExACT	F	10	White British
2	A	Parent ‐ ExACT	F	38	White British
3	A	Participant – Usual Care	F	12	White British
4	A	Parent – Usual Care	F	43	White British
5	A	Participant – Usual Care	F	14	White British
6	A	Parent – Usual Care	F	52	White British
7	A	Participant ‐ ExACT	M	24	White British
8	A	Participant – Usual Care	M	38	White British
9	A	Participant‐ ExACT	F	16	White British
11	B	Participant ‐ ExACT	F	23	White British
12	B	Participant ‐ ExACT	F	11	White British
13	B	Parent ‐ ExACT	F	38	White British
14	B	Participant – Usual Care	M	10	White British
15	B	Parent – Usual Care	F	40	White British
16	B	Participant – Usual Care	M	45	White British
17	A	Decliner	M	40	White – European
18	A	Decliner	M	19	White British
19	B	Decliner	F	45	White British
20	B	Decliner	F	38	White British
10	A	Drop Out ‐ ExACT	F	24	White – European
21	A	Pediatric CF Nurse	F	49	White British
22	A	Pediatric Physiotherapist	F	43	White British
23	A	Adult Research Nurse	F	55	White British
24	A	Pediatric Physiotherapist	F	34	White British
25	A	Adult Consultant	F	57	White British
26	A	Research Nurse	F	59	White British
27	A	Adult Physiotherapist	F	54	White British
28	B	Pediatric Research Nurse	F	41	White British
29	B	Pediatric Physiotherapist	M	43	White British
30	B	Adult Physiotherapist	F	26	White British
31	B	Pediatric Research Nurse	F	29	White British
32	B	Pediatric Research Nurse	F	55	White British

Abbreviations: CF, cystic fibrosis; CTAP, UK Cystic Fibrosis Trust Clinical Trials Accelerator Platform; ExACT, exercise as an airway clearance technique; F, female; M, male; MDT, multidisciplinary team.

**TABLE 2:** Theme 1: Experiences of and lessons learnt from the ExACT‐CF study.

Sub‐theme	Facilitators	Barriers
1.1 Recruitment	Clear recruitment materials Positive attitudes towards physical activity Desire for evidence base Involvement in previous studies Study not anticipated to be onerous	Unwilling to be randomized due to strong personal preferences Recruitment within a busy clinic setting Other competing studies Personal circumstances Geographical location
1.2 Randomization	Already doing exercise	Preference for specific study arm
1.3 Day‐to‐day running and participation in the study	Supportive research team Familiarity with study questionnaires Clear and concise information sheets about trial arm Ability to fit intervention into daily routine Ease of data collection and reporting Flexibility of study visit and weekly call timings	Getting into initial routine Dose and frequency of intervention on a long‐term basis Accuracy of retrospective data Database rigidity Technical issues

**TABLE 3:** Theme 2: Towards choice and flexibility: Evolving perspectives on airway clearance.

2.1. Views around traditional physiotherapy (chest physiotherapy)	Considered boring and time consuming Not as important (“feeling better” due to new treatments) Low adherence with usual care Resulting demoralization
2.2. Views around exercise as an airway clearance technique	Convenient, easy and time‐saving Safe and effective Greater enjoyment and motivation, feeling ‘normal’ Variability and choice Less burdensome for parents Weather dependent Challenging at times to fit into daily routines Limited during injury or exacerbation Hesitation from clinicians to prescribe (due to lack of clinical evidence)
2.3. ‘There's a case for both at differing times’	Need for more safety evidence to enable informed patient decision Recommendations and guidance (step down and interchange model) needed Personalized choices based on individual circumstances and preferences Education needed (stratifying how much exercise equates to usual care session, required frequency and recognizing exacerbation)

### THEME 1: Experiences of and Lessons Learnt From the ExACT‐CF Study

This theme captures perspectives on recruitment (1.1), randomization (1.2), and study participation and trial procedures (1.3), highlighting both facilitators and barriers to feasibility.

### 1.1: Recruitment

**Facilitators** included clear information sheets, straightforward consent processes, and positive relationships with familiar clinical and research staff, including who participants were approached by. Motivation to reduce treatment burden, potential to change future treatment options and interest in exercise made the trial appealing. Some viewed participation as a way to trial a preferred alternative and reduce reliance on chest physiotherapy. The low number of face‐to‐face visits and use of virtual check‐ins also supported engagement:

The good thing is there were not a lot of visits, and that's what puts me off trials; is if it involves a lot of coming to the hospital.

(Male, 45 years, Participant, Site B)

Although target recruitment numbers were met, there were multiple challenges. **Barriers** included strong pre‐existing treatment preferences. Some pwCF were reluctant to risk being randomized into the opposite of their usual practice, either avoiding restarting chest physiotherapy or stopping chest physiotherapy completely. Three of the four decliners admitted not doing regular chest physiotherapy and did not want to have to restart (if allocated to usual care), because “*that wouldn't probably be comfortable for me”* (*Male, 40 years, Decliner, Site A*); whereas the fourth stated they would not feel comfortable stopping their chest physiotherapy and replacing it with exercise completely.

I've done Physio since I was 2, I don't know what would make me less nervous about stopping chest PT but I am not comfortable with oh, well, you know, let's see whether this works.

(Female 38 years, Decliner, Site B)

Similarly, staff reported that many pwCF had already replaced traditional chest physiotherapy with exercise or were not willing to stop traditional chest physiotherapy, “*they already stopped airway clearance so the thought of going back onto it would have been awful*” (Female 40 years, Pediatric Physiotherapist, Site A).

Others cited research fatigue due to multiple ongoing studies. Four trial staff highlighted that the multitude of competing studies that pwCF were being approached about posed a challenge with the potential risk of study saturation and fatigue: *“At first we had quite a lot of our patients, in the age bracket, on another trial, so we had to wait for a lot of them to come back to us”* (*Male, 43 years, Pediatric Physiotherapist, Site B*). To attract more participants and minimize them being overwhelmed at busy clinic appointments, suggested improvements included earlier access to recruitment materials (e.g., posters, leaflets, and social media), allowing more time for the information to be digested: “*so it's not just being completely sprung on them when they're a little bit overwhelmed with everything else”* (*Female, 26 years, Physiotherapist, Site B*).

Personal circumstances, such as financial implications (e.g., not paid when not at work for trial visits), and juggling work and family life, holidays, exams, childcare, were also mentioned as potential barriers to taking part by both participants and healthcare staff. Having more sites open for study visits was suggested by three healthcare professionals and one decliner, to reduce travel burden.

### 1.2: Randomization

**Facilitators:** Several healthcare professionals noted that participants did not seem concerned about being randomized or which trial arm they had been assigned, which was also reflected in some of the participant feedback, “…*it didn't really bother me which group I would be randomized into”* (*Male, aged 45 years, Participant, Site B*).

Most participants reported that undertaking exercise was already a part of their normal day.

**Barriers:** On reflection, some participants expressed clear preferences post‐randomization, displaying emotions ranging from being “*disappointed”* (*Male, 10 years, Participant, Site B*) to “*quite relieved”* (*Female, 14 years, Participant, Site A*).

### 1.3: Study Participation and Trial Procedures

Overall participants reported a very positive experience. **Facilitators** included efficient appointment scheduling, flexible communication, and a friendly, approachable, and supportive research team. Most participants felt there was enough information given about their trial arm post‐randomization, reporting that the outline sheets were clear, concise and had “*everything I needed to know”* (*Female, 10 years, Participant, Site A*). Parent and caregivers also rated the information provided highly ‐ “*I thought it was quite simple and self‐explanatory really”* (*Parent, aged 38 years, Site B*). Participants generally found the intervention duration manageable and not too burdensome. The majority (5/7) of participants and caregivers interviewed who completed the ExACT arm reported that they could easily incorporate daily exercise, with minimal disruption or cost, especially those already active: “*20 minutes once a day was fine…I do running and swimming anyway…it made me feel a lot better running every day”* (*Female, aged 16, Participant, Site A*). Two participants and one study drop out that were interviewed acknowledged finding it a challenge to undertake ExACT every day: “*…during the week it was no problem…the hardest was on the weekend because, I was quite busy some weekends. So although it was only 20 min it was still a bit harder to find the time…I managed to find time 95% of the days”* (*Male, aged 24, Participant, Site A*).

The compendium of exercises was appreciated, including a wide range of enjoyable and convenient exercises to choose from. For those in the Usual Care arm, continuing established routines felt straightforward: “*it was probably the easiest study… that we've done”* (*Female, 40 years, Parent, Site B*).

Prior familiarity with some of the study questionnaires was beneficial, and participants felt they had enough time and support to complete these. Eight participants and parents spoke favorably about the daily diary, describing it as easy to use and liked that it was able to capture mood and be completed retrospectively Participants and parents/caregivers valued seeing physical activity data, which felt: “*quite rewarding… to see how hard she actually does work on a normal day‐to‐day basis”* (*Female, 43 years, Parent, Site A*).

Weekly calls were well received by participants for being short, flexible, and supportive. The ability to complete the final questionnaires online reduced burden and potential transcriber error. Trial staff suggested the need for an approved window for calls and study visits, to minimize protocol deviations and optimize flexibility. Finally, trial staff praised the user‐friendly database and adequate training provision.

***Barriers*** to adherence were more common in participants less accustomed to daily exercise or traditional physiotherapy. Some reported difficulties maintaining daily routines, particularly where exercise was not already embedded in their care or where people in the usual care arm had already replaced their usual care with exercise and were “*out of the habit of doing it daily”* (*Male, 45 years, Participant, Site B*), or had to rush through the physiotherapy and ended up “*cramming it in”* (*Male, 38, Participant, Site A*). While 20 minutes of moderate‐to‐vigorous exercise was generally acceptable, there was recognition that this may not always be feasible, especially on a longer‐term basis, due to work commitments, busy days, holidays, exams, tiredness; “*I think it would be harder for people to commit to doing 20 minutes intense exercise every day for a longer period of time…maybe if it wasn't every day or there was support…it would be more attractive”* (*Male, aged 45, Participant, Site B*). One adult participant suggested that 80% of the time would be more realistic.

Some found data collection (diary, activity watch and study questionnaires) difficult at times, with three participants and one caregiver revealing that they did not complete the diary every day. Although the ability to complete retrospectively was thought beneficial for some participants/caregivers, there was concern that this might influence the accuracy of data, and the reliance on parents for children's entries was noted. Technical issues with wearables (Garmin activity watch), internet stability, and synchronizing platforms for data logging (Garmin activity watch and spirometer) was noted by participants and trial staff. Six adult participants also reported that sometimes they struggled to remember to press the button to record their data on the watch or forgot to charge it reducing the duration and accuracy of data collection.

Regarding some study questionnaires and tests, clarifications and additional education around some of the less familiar questionnaires and study tests were sought, for example, advice if people felt unwell and how to gradually increase activity levels. Increased day‐to‐day collaboration across sites was also mentioned as a potential area for improvement by trial staff, so they “*could have maybe supported and helped each other a bit more”* (*Female, 59 years, Research Nurse, Site A*).

Finally, there were comments around the database rigidity and some technical issues with other data collection methods. Adult participants with CF suggested improvements, such as free‐text diary sections or an additional text box would allow additional comments, such as why they had been unable to complete something or changes to sputum production. Trial staff suggested the ability to part save entries mid‐way and then re‐access to compete data entry at a later stage, to “*pull through*” duplicate data, and the use of consistent wording and formats would ease data collection and transfer and improve efficiency. Two trial staff identified challenges to remote calls, namely devise synchronization and data upload along with reduced child engagement in remote settings.

### THEME 2: Towards Choice and Flexibility: Evolving Perspectives on Airway Clearance

This theme explores perceptions of traditional physiotherapy (2.1), experiences with exercise as an ACT (2.2), and the desire for evidence to support personalized, educated care choices (2.3).

### 2.1: Views Around Traditional Physiotherapy

These data were gained from asking about opinions or preferences for exercise or traditional airway clearance before the trial, and also from experiences within the trial. When asked about opinions or experiences of traditional airway clearance (chest physiotherapy) and exercise before the trial, chest physiotherapy was commonly described as time‐consuming, disruptive, and burdensome. Four health care professionals mentioned that pwCF often reported, to them, that usual care was a time‐consuming chore disturbing their daily routines. Many participants (including three decliners) explained that they had already transitioned away from it, replacing sessions with exercise, a theme also noted by some health professionals:

A lot of our patients with CF already do swap in some of their airway clearance times, if you like, the times they were meant to be doing [traditional] airway clearance for exercise.

(Female, 54 years, Physiotherapist, Site A)

HEMT was seen as a turning point, leading some to deprioritise chest physiotherapy. One adult participant with CF acknowledged that physiotherapy was not as important since starting on modulator combination therapy and five healthcare professionals reported that, for some patients, airway clearance was not a priority, and they had stopped chest physiotherapy. Reflecting on their previous experiences, before the study, participants reported feeling criticized for low adherence and often demotivated when their chest physiotherapy was not effective, despite trying hard and spending significant time doing it, which was also reflected in some of the interviews with healthcare professionals and trial staff, for example:

…it would be nice for the physios to be able to devise a routine for them that includes exercise so that they're not constantly being told they're not adhering, because I think that can be quite damaging.

(Female, 29 years, Research Nurse, Site B)

### 2.2: Views Around Exercise as an Airway Clearance Technique

Trial staff reported that many pwCF were open to try alternatives and that they themselves were keen to advocate exercise. Benefits reported by health care professionals and trial staff, as well as participants and parents of using exercise for airway clearance included: (a) convenience (easy to embed in their lives and time‐saving), (b) safe and effective in producing sputum and improve their wellbeing, (c) an intervention that offers variability of exercise and choices, thus greater enjoyment and motivation to do it, (d) an intervention that is less burdensome for the parents as “*there was none of this 'will you just get this done?’… There was no stress in the morning, that was completely taken away”* (*Female, 38 years, Parent, Site A*), and (e) an intervention that makes them feel “*normal again … they're doing it with friends, so it doesn't feel like it's such a chore”* (*Female, 29 years, Research Nurse, Site B*).

On the other hand, although exercise (ExACT) was considered an acceptable and preferred technique, participants, caregivers and healthcare professionals acknowledged three potential limitations: (a) exercise could carry cost implications because it is weather and season dependent, which may limit outdoor activities (e.g., in the winter months), thus patients would need to buy home equipment or pay a gym membership; (b) it can be challenging at times to fit daily routines due to work, holidays, exams, busy lifestyles, especially for individuals, who did not usually undertake exercise; and (c) exercise is not doable during periods of injury or exacerbation at the required frequency, intensity, and duration.

### 3.1 2.3: “There's a Case for Both at Differing Times”

Most interviewees endorsed an interchangeable model, using both exercise and chest physiotherapy depending on context. While clinicians were enthusiastic about ExACT, some felt constrained by the lack of robust clinical evidence: “*some very purist physios maybe are not on board with it”* (*Female, 22 years, Physiotherapist, Site A*), feeling nervous about reducing the number of sessions of chest physiotherapy, and concerned that pwCF may de‐skill (lose skills of carrying out traditional chest physiotherapy) if not carried out regularly. Although no safety concerns about replacing their chest physiotherapy were reported, one healthcare professional commented that parents may be more apprehensive than their children (e.g., when unwell) and one of the decliners reported being uncomfortable replacing chest physiotherapy with exercise completely.

Healthcare professionals would like to advocate exercise as the way forward because *“for years we've had patients who have asked about it”* (*Female, 22 years, Physiotherapist, site A*), especially for pwCF who may not be currently doing any chest physiotherapy, but it was acknowledged that more clinical evidence and specific guidance is needed “*so they [patients] can make a more informed decision”* (*Female, 34 years, Physiotherapist, Site A*).

I was really pro exercise, and I have just always been really honest with patients and said I can't give an evidence‐based answer to using it instead of airway clearance… right now, we don't have the evidence to say that.

(Female, 34 years, Physiotherapist, Site A)

Suggestions included a step‐down approach (one session replaced at a time), or using an alternating/interchange model, tailored to lung function, fitness, and daily routines. Professionals emphasized the need for education on recognizing exacerbations and maintaining core physiotherapy skills. All healthcare professionals from both sites (including doctors, nurses and physiotherapists) mentioned the importance of pwCF being able to recognize an exacerbation and education around when to restart traditional airway clearance techniques during these times, or when there was not time to exercise, and the importance of maintaining the skills required to carrying out effective chest physiotherapy. The consensus was not to completely replace chest physiotherapy but instead show that, at times, certain exercise with forced expiratory maneuvers could be used for airway clearance by “*doing exercises on certain days… and then airway clearance on the days when you're not doing exercise. So, a nice balance of it”* (*Male, 43 years, Physiotherapist, Site B*).

All participants and parents, following completion of the study, and three of the decliners shared that, going forward, their preference would be to carrying out exercise airway clearance alone, or using a mix of exercise airway clearance and other forms of chest physiotherapy. They discussed that “*there's a case for both at differing times”* (*Male, 45 years, Participant, Site B*), to be used interchangeably complementing each other. Both study participants and healthcare professionals acknowledged the importance of having some flexibility, and “*it would feel great to be able to choose”* (*Female, 10 years, Participant, Site A*) between exercise and other chest physiotherapy ACTs. The interchangeable model would enable pwCF to choose their technique based on individual circumstances and educated choices, taking into consideration various factors (e.g., personal preferences, lung function, age, daily routines lifestyles, fitness levels, symptoms, weather, etc.).

Importantly, no participant advocated for exclusive use of traditional physiotherapy going forward:

“I think I would always prefer exercise over the traditional physio… It feels like something you are choosing to do more than something you have to do… However, I don't think that traditional airway clearance is something that I will ever not do… it will always be something that I can go to if I'm feeling run down, if I'm feeling more congested than normal, I know I can do that.” (Male, 38 years, Participant, Site A).

## 4 Discussion

### 4.1 Key Findings

This is the first qualitative study to explore experiences of pwCF, caregivers, and healthcare professionals participating in, or supporting, a feasibility trial of an exercise‐based airway clearance intervention. Participants and staff described ExACT as an acceptable, feasible, and safe alternative to traditional chest physiotherapy, valued for its flexibility, ability to fit into daily routines, and social benefits. In particular, exercising with friends or family contributed to a sense of normality. Despite minor barriers, such as illness or poor weather, no participant preferred chest physiotherapy alone going forward. Instead, there was a strong appetite for personalized, flexible, and interchangeable airway clearance models ‐ echoing wider calls for individualized care in the HEMT era [17, 20].

Traditional chest physiotherapy was consistently described as time‐consuming, burdensome, and demoralizing; aligning with previous reports [3, 4]. These perceptions, compounded by the transformative effects of HEMT in some pwCF, have led many pwCF to independently substitute or supplement conventional ACTs with exercise [8, 22]. However, both pwCF and healthcare professionals emphasized the need for robust clinical evidence and guidance to support informed decision‐making around ExACT, including clear recommendations on dose, frequency, and safety parameters. This builds nicely on our prior Delphi consensus work, and evidence highlighting that findings to date have been heterogeneous and insufficient to inform practice (e.g., [21]), supporting participants’ calls for larger, definitive evaluations.

Overall, trial procedures were well‐received. Participants and staff found the recruitment, randomization, and data collection processes straightforward. Study participants had a positive experience in their allocated group, although some difficulties with adherence were reported. The completion of diaries, questionnaires and activity monitoring were thought easy, with minimum burden to pwCF and, where relevant, caregivers. Support and communication with the study team were positively noted. Several facilitators to participation in future trials were identified, including previous research involvement, positive attitudes toward exercise and clinical evidence, ongoing incentives, and flexibility in allocation and intervention dose. Challenges included technical issues, staffing capacity, and the need for more adaptable data entry tools (e.g., adding more choices to the diaries and databases). Addressing these practical concerns will be key to optimizing a future definitive trial.

### 4.2 Study Strength and Limitations

A major strength of this study lies in its novelty. Indeed, it is the first to qualitatively evaluate an exercise‐based airway clearance intervention in pwCF within a clinical trial context, aiming to replace or complement traditional chest physiotherapy with exercise. Grounded in the theoretical framework of acceptability [32], the study captured perspectives from pwCF, caregivers, and healthcare professionals, providing in‐depth insight into intervention acceptability, trial feasibility, as well as the perceived value of ExACT as a therapeutic option for airway clearance. Extensive patient and public involvement and engagement (PPIE) shaped the design, recruitment, and dissemination strategies for this trial, enhancing its relevance and responsiveness to community needs. However, PPIE was not included in the analysis phase due to resource constraints, which may have limited interpretive nuance. The sample included a range of ages, genders, and roles, but lacked a range of ethnic diversity that would be reflective of UK CF demographics. Some interviews were time‐limited due to clinical pressures; however, they remained focused and informative. Finally, as a short‐term feasibility study (28 days), findings may not fully represent long‐term adherence challenges or benefits.

### 4.3 Relation to Current Evidence

Our findings align with recent updates to the European CF Standards of Care, which endorse a more flexible, preference‐based approach to ACTs, including exercise as part of a broader airway clearance toolkit [20]. Reducing the treatment burden for pwCF remains a key research priority [9, 33], and this study supports ExACT as a potentially viable, lower‐burden alternative to chest physiotherapy. Participants consistently preferred ExACT either alone or in combination with traditional techniques, reinforcing previous reports of the importance of aligning ACTs with individual preference, perceived benefit, and compatibility with lifestyle [34]. This study adds important context by exploring these experiences within a clinical trial setting. Participants reported greater satisfaction and motivation with ExACT, suggesting that adherence may be improved when interventions are personalized, meaningful, and embedded in everyday life. The findings underscore the need to move beyond a “one‐size‐fits‐all” approach and offer diverse, evidence‐based options that pwCF can tailor to their needs [34].

### 4.4 Implications for Clinical Practice

Adherence to chest physiotherapy remains a significant challenge in real‐world settings. For example, Filipow et al. [35] reported that just 20.7% of chest physiotherapy sessions in children and young people with CF met prescribed standards, with some patients averaging fewer than one effective session every 6 weeks. Despite best intentions, traditional ACTs may therefore offer limited clinical benefit when poorly implemented. In contrast, participants in our study described ExACT not only effective in promoting sputum clearance, but also as more sustainable and enjoyable, with promising short‐term (28 days) adherence outcomes [26].

The introduction of CFTR modulators has shifted the landscape of CF care, with many pwCF reporting reduced sputum production [14, 15, 36] and feeling “normal” for the first time. As a result, some individuals have independently reduced or replaced chest physiotherapy with exercise, at least some of the time [8, 22]. While this reflects the evolving lived experience of pwCF, physiotherapists remain understandably cautious about recommending exercise as a primary airway clearance technique without robust evidence of its safety and efficacy [21]. Chest physiotherapy continues to be reported as burdensome, time‐consuming and, when poorly implemented [35], potentially ineffective, prompting calls to reassess long‐standing treatment paradigms in light of improved health status and life expectancy. Our findings suggest that ExACT may offer a flexible, patient‐centered alternative to conventional airway clearance, particularly when supported by appropriate clinical guidance and ongoing feedback. Integration of ExACT into standard care could help improve adherence, reduce treatment fatigue, and enhance quality of life. However, further research is required to establish its efficacy and long‐term acceptability.

### 4.5 Unanswered Questions and Future Research

There is a clear call from pwCF, caregivers, and healthcare professionals for a definitive, adequately powered trial of ExACT. This trial should address critical uncertainties around clinical efficacy, safety and, subsequently, implementation into CF care pathways, building on the lessons learned from this feasibility study [25, 26]. In doing so, it will not only support the clinical community in making evidence‐informed recommendations but also respond directly to the needs of pwCF who frequently find conventional ACTs unmanageable. It should also prioritize meaningful co‐design, inclusivity, and real‐world relevance, ensuring engagement of pwCF from diverse backgrounds, geographical settings, and disease severities to design airway clearance strategies that are both effective and equitable.

ExACT has the potential to deliver dual benefits: supporting respiratory health and general health and wellbeing, while reducing the significant treatment burden that pwCF face. Its relevance may also extend to other chronic suppurative lung diseases, such as bronchiectasis and primary ciliary dyskinesia. As exercise‐based interventions, such as ExACT, become increasingly integrated into the management of pwCF, ongoing factors recognized within both trial arms, such as difficulty in embedding and maintaining routines, especially over longer time periods, must be addressed. Future research should explore strategies to enhance multidisciplinary support for pwCF, including the integration of behavior change approaches, collaborative working, and improved access to resources, such as clinical exercise physiologists [37, 38], physiotherapists, and community‐based facilities. These supports could play a vital role in maintaining adherence and maximizing long‐term benefit.

## Author Contributions

Conceptualization: Emily Taylor, Dia Soilemezi, Don S. Urquhart, Steve Cunningham, Steff Lewis, Aileen Rae Neilson, Hannah Ensor, Ioannis Vogiatizis, Lorna Allen, Zoe L. Saynor. Data curation: Emily Taylor, Dia Soilemezi, Don S. Urquhart, Zoe L. Saynor. Formal analysis: Emily Taylor, Dia Soilemezi, Don S. Urquhart, Zoe L. Saynor. Funding acquisition: Dia Soilemezi, Don S. Urquhart, Steve Cunningham, Steff Lewis, Aileen Rae Neilson, Hannah Ensor, Ioannis Vogiatizis, Lorna Allen, Zoe L. Saynor. Investigation: Emily Taylor, Dia Soilemezi, Don S. Urquhart, Zoe L. Saynor. Methodology: Emily Taylor, Dia Soilemezi, Don S. Urquhart, Steve Cunningham, Steff Lewis, Aileen Rae Neilson, Hannah Ensor, Ioannis Vogiatizis, Lorna Allen, Zoe L. Saynor. Project administration: Emily Taylor, Dia Soilemezi, Don S. Urquhart, Zoe L. Saynor. Resources: Emily Taylor, Dia Soilemezi, Don S. Urquhart, Lorna Allen, Zoe L. Saynor. Supervision: Dia Soilemezi, Don S. Urquhart, Zoe L. Saynor. Validation: Emily Taylor, Dia Soilemezi, Don S. Urquhart, Steve Cunningham, Steff Lewis, Aileen Rae Neilson, Hannah Ensor, Ioannis Vogiatizis, Lorna Allen, Zoe L. Saynor. Visualization: Emily Taylor, Dia Soilemezi, Don S. Urquhart, Steve Cunningham, Steff Lewis, Aileen Rae Neilson, Hannah Ensor, Ioannis Vogiatizis, Lorna Allen, Zoe L. Saynor. Writing – original draft: Emily Taylor, Dia Soilemezi, Don S. Urquhart, Zoe L. Saynor. Writing – review and editing: Emily Taylor, Dia Soilemezi, Don S. Urquhart, Steve Cunningham, Steff Lewis, Aileen Rae Neilson, Hannah Ensor, Ioannis Vogiatizis, Lorna Allen, Zoe L. Saynor.

## ExACT‐CF Study Group

Don S. Urquhart (Department of Child Life and Health, University of Edinburgh, Edinburgh, UK; Department of Pediatric Respiratory and Sleep Medicine, Royal Hospital for Children and Young People, Edinburgh, UK); Emily Taylor (Department of Child Life and Health, University of Edinburgh, Edinburgh, UK; Department of Pediatric Respiratory and Sleep Medicine, Royal Hospital for Children and Young People, Edinburgh, UK); Steve Cunningham (Department of Child Life and Health, University of Edinburgh, Edinburgh, UK; Department of Pediatric Respiratory and Sleep Medicine, Royal Hospital for Children and Young People, Edinburgh, UK; Centre for Inflammation Research, University of Edinburgh, Edinburgh, UK); Steff Lewis (Usher Institute, University of Edinburgh, Edinburgh Clinical Trials Unit, NINE Edinburgh Bioquarter, 9 Little France, Edinburgh, UK); Aileen Rae Neilson (Usher Institute, University of Edinburgh, Edinburgh Clinical Trials Unit, NINE Edinburgh Bioquarter, 9 Little France, Edinburgh, UK); Dia Soilemezi (Department of Psychology, Faculty of Science and Health, University of Portsmouth, Portsmouth, UK); Hannah Ensor (Usher Institute, University of Edinburgh, Edinburgh Clinical Trials Unit, NINE Edinburgh Bioquarter, 9 Little France, Edinburgh, UK); Ioannis Vogiatizis (Department of Sport, Exercise and Rehabilitation, Faculty of Health and Life Sciences, Northumbria University Newcastle, UK); Lorna Allen (Cystic Fibrosis Trust, One Aldgate, Second Floor, London, UK); Debbie Miller (Adult Cystic Fibrosis Unit, Western General Hospital, Edinburgh, United Kingdom); Donna Bowen (NIHR Southampton Clinical Research Facility, University Hospitals Southampton NHS Foundation Trust, Southampton, United Kingdom); Ellyse Kilarski (Adult Cystic Fibrosis Unit, Western General Hospital, Edinburgh, United Kingdom); Ellen Lacey (NIHR Southampton Clinical Research Facility, University Hospitals Southampton NHS Foundation Trust, Southampton, United Kingdom); Gary Connett (National Institute for Health and Care Research, Southampton Biomedical Research Centre, University Hospital Southampton NHS Foundation Trust, UK; Department of Pediatric Respiratory Medicine, University Hospital Southampton NHS Foundation Trust, Southampton, United Kingdom); Julian Legg (Department of Pediatric Respiratory Medicine, University Hospital Southampton NHS Foundation Trust, Southampton, United Kingdom); Hazel Evans (Department of Pediatric Respiratory Medicine, University Hospital Southampton NHS Foundation Trust, Southampton, United Kingdom); Thomas Daniels (Wessex Cystic Fibrosis Unit, University Hospitals Southampton NHS Foundation Trust, Southampton, UK; National Institute for Health and Care Research, Southampton Biomedical Research Centre, University Hospital Southampton NHS Foundation Trust, UK); Mark Allenby (Wessex Cystic Fibrosis Unit, University Hospitals Southampton NHS Foundation Trust, Southampton, UK); Mary Carroll (Wessex Cystic Fibrosis Unit, University Hospitals Southampton NHS Foundation Trust, Southampton, UK); Robert Gray (Adult Cystic Fibrosis Unit, Western General Hospital, Edinburgh, United Kingdom); Carlos Echevarria (Adult Cystic Fibrosis Unit, Royal Victoria Infirmary, Newcastle‐upon‐Tyne, United Kingdom); Lisa Morrison (West of Scotland Adult CF Unit, Queen Elizabeth University Hospital, Glasgow, United Kingdom); Gordon McGregor (West of Scotland Adult CF Unit, Queen Elizabeth University Hospital, Glasgow, United Kingdom); Mary Abkir (Royal Brompton & Harefield Hospitals, part of Guys and St Thomas’ NHS Foundation Trust, London, United Kingdom; National Heart and Lung Institute, Imperial College London, London, United Kingdom; European Cystic Fibrosis Society, Lung Clearance Index Core Facility, London, United Kingdom); Clare Saunders (Royal Brompton & Harefield Hospitals, part of Guys and St Thomas’ NHS Foundation Trust, London, United Kingdom; National Heart and Lung Institute, Imperial College London, London, United Kingdom; European Cystic Fibrosis Society, Lung Clearance Index Core Facility, London, United Kingdom); Zoe L. Saynor (School of Health Sciences, Faculty of Environmental and Life Sciences, University of Southampton, Southampton, UK; Wessex Cystic Fibrosis Unit, University Hospitals Southampton NHS Foundation Trust, Southampton, UK; National Institute for Health and Care Research, Southampton Biomedical Research Centre, University Hospital Southampton NHS Foundation Trust, UK).

## Disclosure

The views expressed are those of the author(s) and not necessarily those of the NIHR or the Department of Health and Social Care.

## Conflicts of Interest

The authors declare no conflicts of interest.

## Supporting information


**Table S1:** Additional illustrative quotes for Theme 1: Experience of and lessons learnt from the ExACT‐CF study. **Table S2:** Additional illustrative quotes from Theme 2: Towards choice and flexibility: evolving perspectives on airway clearance.

## Data Availability

All data generated or analyzed during the current study are available from the Principal Investigators of the study (Z.L.S. and D.S.U.) on reasonable request.
