# Supplementary material for: Exploring Exercise as Airway Clearance in Cystic Fibrosis: A Qualitative Study From the ExACT‐CF Feasibility Trial
Source: Pediatr Pulmonol. 2026 Jan 26;61(1):e71470. doi: 10.1002/ppul.71470 (PMC12834544; doi:10.1002/ppul.71470)
Supplement: Supplementary file 1 — Table S1: Additional illustrative quotes for Theme 1: Experience of and lessons learnt from the ExACT‐CF study. Table S2: Additional illustrative quotes from Theme 2: Towards choice and flexibility: evolving perspectives on airway clearance. [file PPUL-61-0-s001.docx]

**ONLINE SUPPLEMENTARY MATERIAL**

**Table S1.** Additional illustrative quotes for Theme 1: Experience of and lessons learnt from the ExACT-CF study.

| **Subthemes** | | **Additional illustrative quotes** |
| --- | --- | --- |
| **Recruitment** | Clear Information leaflets | *“[The]* *leaflet was pretty thorough, as well as the information from the doctor. He was pretty clear on what was involved over the course, appointment-wise and content-wise, so there was no surprises or anything that was unexpected” (John, 24 years, Participant, ExACT, Site A).*  *‘everything was quite self-explanatory in the information that we had, the handouts were good.’ (Female, 43 years, Parent, Usual Care, Site A).*  *‘I thought the information leaflet was pretty well put together.’ (Male, 38 years, Participant, Usual Care, Site A).*  *‘they’re really good with all the information they give you’ (Female, 40 years, Parent, Usual Care, Site B).* |
|  | Positive attitudes towards physical activity and research | *‘I think that I’ve been personally very lucky with the way that my treatment for CF has developed in my lifetime, and I’ve always felt like anything that I could kind of give back, so that maybe other people who are born in the future with CF have an easier time, that’s always been a worthwhile cause for me’ (Male, 38 years, Participant, Usual Care, Site A).*  ‘Well, we initially thought that if it can improve, our daily routine, by doing less, then it was definitely a good thing and potentially future as well for other people. And because I suppose that she is so energetic and does so much exercise, we felt that it wouldn’t be any more work for her – it was something that she done on a daily basis anyway.’ (Female, 43 years, Parent, Usual Care, Site A). |
|  | Involvement in previous studies and familiarity with staff | *‘I think there’s also an element of who asks them and how you ask them. If they’ve got a positive relationship with the person who asks them, and you ask them in a way that you know that they’ll be inclined towards supporting you, then you’re going to get more people being involved. If I ask a person to be involved in a specific way, and a doctor asked them to be involved in the same study but not the same way as I would put it, they might do it for me but not for him. So, it really depends sometimes. It’s on the relationship you have with the person when you ask them, and also what they hope to get out of it, I think’ (Female, 54 years, Physiotherapist (adult care), Site B).*  *‘Having been in a study since 2020, we were like, what’s one more… there was no real downside for me. I was like, well, whichever group he is in, it’s not going to have a negative impact on his health, and on his care. Like I’ve said, we’ve grown quite attached to XX and the team, and just why not? (Female, 40 years, Parent, Usual Care, Site B).* |
|  | Potential to not have to do traditional ACT’s | *‘I wanted to join it because felt like that it would be like an amazing thing to start because it’s like it means I don’t have to use the aerobica for a month which is amazing because it’s quite boring needing to do something every single day really of your life but changing it up means it’s quite fun … .....it’s amazing because you could change like lots of stuff for other people who don’t like doing the aerobica’ (Female, 10 years, Participant, ExACT, Site A).* |
|  | Minimal study visits | *The good thing is there were not a lot of visits, and that’s what puts me off trials; is if it involves a lot of coming to the hospital’ (Male, 45 years, Participant, Usual Care, Site B).* |
|  | Unwillingness to be randomised due to strong individual preferences | *‘The reason I decided not to participate, the main reason was that I am currently not really doing any regular form of physiotherapy because since I started taking the Kaftrio I basically stopped coughing up anything so there’s no sputum anymore in my everyday life so the reason for that is that I don’t do a regular physiotherapy at home right now. And, as far as I was able to read in the materials that I received, is that it is not possible for the participant to choose the exact group that I will be put into so it’s totally random whether I would be in a group that does the physical exercise or whether I’m in a group that does the physiotherapy, so that’s why I thought well if I get randomly selected to the group that’s supposed to do the physiotherapy as normal that wouldn’t probably be comfortable for me.’(Male, 40 years, Decliner, Site A).*  *‘to be honest, it probably was to do with the airway clearance and randomisation ..because exercise, you know, I'm good. I go to the gym and I do quite well. But airway clearance there is not a hope in hell (laughs).’ (Female, 38 years, Decliner, Site B).*  *‘We’ve got a lot of people now who don’t do much airway clearance and tend to use exercise as their airway clearance… a lot of them were just like, no, I’m not going back to doing that.’ (Female, 26 years, Physiotherapist, Site B).*  *‘patients who are on (CFTR) modulators, are feeling so much better, not producing so much. So, I think it’s another thing that’s just slipped because they’re feeling a bit better. So, then that made it quite difficult to recruit, because they were worried that they were going to have to be in the physiotherapy group and then they would have to start that again’ (Female, 59 years, CTAP Nurse, Site B).*  *‘I think all the ones that would have done brilliantly were almost self-selected for exercise anyway. You know, they already stopped airway clearance so the thought of going back onto it would have been awful’. (Female, 43 years, Physiotherapist (paediatric care), Site A).* |
|  | Geographical locations and personal circumstances. | *‘basically, it's just the time thing, every time I've got an appointment at the hospital I am kind of stressed about getting there and everything. And I just thought I can't add;* *at the moment I'm really busy with work and childcare…. I'm trying to juggle financially …I know you refund the cost. But it's the time I get paid when I'm at my desk and not when I'm not’. (Female, 45 years, Decliner, Site B).* |
|  | Competing studies | *‘for a lot of people, the first time they’re hearing about it is us asking them in clinic…those appointments can be very long... and they’ve got lots of people to see….If they were aware of it… when they come to clinic, they might already have some idea about it … so it’s not just being completely sprung on them when they’re a little bit overwhelmed with everything else.’ (Female, 26 years, Physiotherapist, Site B).* |
| **Randomisation** |  | Didn’t mind:  ‘*I didn’t mind which group I was in’ (Female, 12 years, Participant, Usual Care, Site A).*  *‘I didn’t mind really but I wasn’t sure if I liked the idea of exercising everyday but … I don’t mind. I didn’t have a strong opinion’ (Female, 14 years, Participant, Usual Care, Site A).*  *‘it didn’t really bother me which group I would be randomised into’ (Female, 45 years, Participant, Usual Care, Site B).*  Hoping for:  *‘I was hoping I was gonna go into the exercise one.’ (Female, 10 years, Participant, ExACT, Site A).*  *‘I really hoped it would be exercise, purely because she does the Aerobica so having the chance to do something different that she’s done for many years now really, really was a great chance to try something completely different and I knew how much she really wanted to be in the exercise group.’ (Female, 38 years, Parent, ExACT, Site A).*  ‘*Yeah, I preferred exercise*.’ *(Female, 16 years, Participant, ExACT, Site A).*  When allocated:  ‘*Happy. Maybe a sense of relief that if I had been put in the physio one I would have four weeks of something that I probably wouldn’t have enjoyed as much as exercise. So, yeah, I was happy to be in the exercise arm.’* *(Male, 24 years, Participant, ExACT, Site A).*  *‘I was quite relieved, because I wasn’t sure if I wanted to do exercise every day or what.’ (Female, 14 years, Participant, Usual Care, Site A).*  *‘I think he was hoping to be in the group that didn’t have to do the physio, and had to do exercise, so I think that was… He was very much, oh, I really want to be in the exercise group.’ (Female, 40 years, Parent, Usual Care, Site B).*  *‘I was kind of disappointed when I was randomised in the UC group’ (Male, 10 years, Participant, Usual Care, Site B).* |
| **Day-to-day participation in and running of the trial** | Adherence to assigned treatment arm - ExACT | *‘I think my preferred was definitely cycling, but at the start I tried to vary it a little bit with a little bit of running, a little bit of swimming, but I think I just fell into the habit of doing the cycling because it was the one that I enjoyed the most. And it was sort of most convenient for me’. (Male, 24 years, Participant, ExACT, Site A).*  *‘a wide variety of things to choose from’ (Female, 38 years, Parent, ExACT, Site A).*  *‘I was able to fit all the sessions in and it didn't disrupt my routine’ (Female, 10 years, Participant, ExACT, Site A).*  *‘It was great to see her happy doing something that was making her fit and well and active. It was amazing. ..’ (Female, 38 years, Parent, ExACT, Site A).*  *‘I was able to fit in 20 minutes every day into my daily routine okay’ (Female, 16 years, Participant, ExACT, Site A).*  *‘It frees up my mornings and afternoons’ (Female, 11 years, Participant, ExACT, Site B).*  *‘20 minutes once a day was fine..I do running and swimming anyway…it made me feel a lot better running every day’ (Female, 16 years, Participant, ExACT, Site A).*  *Challenges:*  *‘…during the week it was no problem…the hardest was on the weekend because, I was quite busy some weekends. So although it was only 20 minutes it was still a bit harder to find the time…I managed to find time 95% of the days’ (Male, 24 years, Participant, ExACT, Site A).*  *‘Yes, and no, for the majority of parts it was manageable…but then there were other days, obviously like if I was working all day, for example, and I like got home, and I was just too-too tired- like too tired…I just couldn’t quite manage it’ (Female, 23 years, Participant, ExACT, Site B).*  *I think it would be harder for people to commit to doing 20 mins intense exercise every day for a longer period of time..maybe if it wasn’t every day or there was support…it would be more attractive’.(Male, 45 years, Participant, Usual Care, Site B*). |
|  | Adherence to assigned treatment arm – Usual Care | ‘*all-in-all it was probably the easiest study out of the two that we’ve done*’ *(Female, 40 years, Parent, Usual Care, Site B).*  *Challenges:*  *‘I did change what I would usually do, even though in the usual care arm .... a bit like those that were on the exercise arm, probably for the first two, three weeks, I was religiously trying to do twice a day on the breathing, but that it probably did tail off, in all honesty’ (Male, 45 years, Participant, Usual Care, Site B).*  *‘I probably didn’t do 100% of the airway clearance I was ‘supposed’ to do on the study’(Male, 45 years, Participant, Usual Care, Site B).*  ‘*out of the habit of doing it daily’(Male, 45 years, Usual Care, Participant, Site B).*  ‘had to rush through his physiotherapy and ended up ‘*cramming it in’* *(Male, 38 years, Participant, Site A).* |
|  | Study information sheets | The information sheets had *‘everything I needed to know’ (Female, 10 years, Participant, ExACT, Site A).*  *‘I thought it was quite simple and self-explanatory really, what the aim was, and what would be involved. I think it’s backed up with you having a conversation with a clinician. And even I think even if I just got the leaflet on its own, without anyone explaining, I would have understood exactly what they wanted, or were looking for.’ (Female, 38 years, Parent, ExACT, Site B).* |
|  | Completion of daily diary | *‘… because you’re just used to doing it and then it felt like, it showed you … for her dad seeing what was involved also it was like ‘wow’ you know, so that’s quite a lot fitting in one day… it was quite rewarding, I think, for us to see how hard she actually does work on a normal day-to-day basis’ (Female, 43 years, Parent, Usual Care, Site A).*  *‘There was some days when I didn’t do it bang on the day, but purely because I fell asleep or I got busy and I did forget about it’ (Female, 38 years, Parent, ExACT, Site A).*  *‘I did like the fact that when I couldn’t record it exactly on the day every day if I went back a day or two I knew how to pull out the stats on what she had done, so I didn’t feel like there was any of them missing.’(Female, 38 years, Parent, ExACT, Site A).*  *Challenges:*  *‘My mum filled in the daily diaries, she just asked me what I had done’ (Female, 14 years, Participant, Usual Care, Site A).*  *‘Yes, she [mum] mainly did it... I was with her when she was doing it’ (Female, 11 years, Participant, ExACT, Site B).*  *‘the diary I didn’t usually do it every day, I’d sort of do it every three days or … put three days in the one shot’ (Male, 24 years, Participant, ExACT, Site A).*  *‘I was missing several days logging in the diary and then having to catch up all in one long session.’ (Female, 16 years, Participant, ExACT, Site A).*  *‘I filled out the diary, the only thing that it didn’t allow for was just normal general illness. So, obviously when it said you didn’t do physio, it was all related to CF stuff, there wasn’t like, actually I was unwell with a normal illness…obviously when she was unwell, reason why we didn’t do physio was because of a normal illness, not CF related, so that was the only thing I picked up that probably should have been accounted for, that it’s not always CF related illnesses, they do obviously get other illnesses.’ (Female, 16 years, Participant, ExACT, Site A).* |
|  | Wearability / usability of Garmin | *‘Garmin watch uploaded itself’ (Female, 38 years, Parent, ExACT, Site A).*  *‘I liked having it on my wrist...it was quite easy’ (Female, 12 years, Participant, Usual Care, Site A).*  *‘The watch wearing was fine’ (Male, 24 years, Participant, ExACT, Site A).*  *‘I loved the watch… the thing I enjoyed the most is actually being able to see the live results, if that makes sense like off the watch’ (Female, 23 years, Participant, ExACT, Site B).*  *‘I got used to it really quick’ (Female, 11 years, Participant, ExACT, Site B).*  *‘And a lot of them liked seeing on the Garmin what they’ve done’ (Female, 29 years, Paediatric Research Nurse, Site B).*  *‘I think the Garmin’s so much better than the other ones.’ (Female, 43 years, Paediatric Physiotherapist, Site A).*  *Challenges:*  *‘Difficulty remembering to start the watch thing. Sometimes I’d start my Arobica and then I’d have to go back and do it’ (Female, 14 years, Participant, Usual Care, Site A).*  *‘There was a couple of times where we went away for a weekend, two weekends, and her watch died. But she did still do exercise, but unfortunately the watch just didn’t log it.’ (Female, 38 years, Parent, ExACT, Site B).*  *‘The app was on my phone … I was having to sync it with my phone. Whereas, because her phone’s with her, it would have synced all the time’ (Female, 38 years, Parent, ExACT, Site B).*  *‘they suggested the app be on my phone, but that meant almost there’s that reliance on me, because of her age, which I get. But obviously because I’d set it up, and it was the parent, it had to be done by me.’ (Female, 38 years, Parent, ExACT, Site B).*  *‘there’s been a few occasions I’ve had to message the team, and go, I have filled it out, why am I getting the text message to say I haven’t... So, that made a little bit more work for me’ (Female, 38 years, Parent, ExACT, Site B).* |
|  | Study Questionnaires | *‘I thought that they were relevant. Having taken part in other studies I was used to filling out some of them. Like the wellbeing side of it. The one that I found the most difficult was breaking down my day into percentages of when I was most active. I think it was slightly disappointing reflecting on how inactive I am for most of the day just because of my job and the way that things play out.’ (Male, 38 years, Participant, Usual Care, Site A).*  *‘Completed all on paper - face to face as sometimes I didn't really understand them. I was given time to complete them all.’ (Female, 16 years, Participant, ExACT, Site A).*  *‘There were a lot of questionnaires...they are used to doing questionnaires, because obviously they do them in clinic anyway, so none of them were surprised by the questionnaires’ (Female, 29 years, Paediatric Research Nurse, Site B).*  *‘There never seemed to be an issue with them not being completed. Certainly by the adult section that I was dealing with.’ (Female, 43 years, Paediatric Physiotherapist, Site A).*  *‘easier when they are in the digital format they can do it before they come; saves a lot of duplication of data... and potential error between what I can see on a piece of paper and what I have typed in.’ (Female, 55 years, CTAP Nurse Adults, Site A).* |
|  | Lung Function Tests | *‘I had never done it before. So the first one was quite a lengthy one’ (Male, 45 years, Participant, Usual Care, Site B).*  *‘The place was familiar, the tests that she had to do, like the LCI, it was all familiar’ (Female, 38 years, Parent, ExACT, Site B).*  *‘I’m used to doing the LCIs, so I felt reasonably comfortable and confident doing it. Yes, no, I thought it went okay, but I do have a lot of experience...I know that it isn’t an easy technique. And new people learning it just need quite a lot of support.’ (Female, 59 years, CTAP Nurse, Site B).* |
|  | Study Database | *‘I think it’s quite user friendly’ (Female, 55 years, CTAP Nurse Adults, Site A).*  *‘Compared to a lot of studies, it was much better, I would say. Easy. Quite user friendly’ (Female, 29 years, Paediatric Research Nurse, Site B).*  *Challenges:*  *‘… it doesn’t save if you haven’t put in spirometry results. That’s a bit annoying, because you’ve put in all that work and then you can’t save it…I can’t save it and if I close it down, I’m going to lose all that information, but I’m waiting on an email to come through from a patient’ (Female, 41 years, Paediatric Research Nurse, Site B).*  *‘I’ve inputted data but I haven’t inputted the spirometry results for a patient. And it doesn’t save if you haven’t put in spirometry results. That’s a bit annoying, because you’ve put in all that work and then you cant save it…I can’t save it and if I close it down, I’m going to lose all that information, but I’m waiting on an email to come through from a patient’ (Female, 41 years, Paediatric Research Nurse, Site B).*  *‘When putting in the new patients and I would put all the data in whilst they were going to have their LCI, but then of course I then had to keep them open because I couldn’t save it until they had done their spirometry..then the fact I couldn’t save it was like oh, okay. So yes, that was frustrating. .. Often diary link would not send and would have to be resent..’ (Female, 55 years, Paediatric Research Nurse, Site B).*  *‘It would be really helpful if, you know, you add the email address once and it pulls through rather than having to add it in numerous places. I just also think there is more error for transcription errors’ (Female, 59 years, CTAP Nurse, Site B).*  *‘It would be helpful if you can save it and then come back to it…Because you don’t always have half an hour to sit down and complete the whole thing in at once.’ (Female, 41 years, Paediatric Research Nurse, site B).*  *‘Sometimes it’s nice to have a free textbox, just because they’ll say stuff that you’re like, doesn’t really fit anywhere’ (Female, 29 years, Paediatric Research Nurse, Site B).*  *‘Being able to enter data and save before complete’ (Female, 55 years, Paediatric Research Nurse, Site B).* |
|  | Study visits  Weekly calls | *‘if we had any questions, we just, cause we was seeing you regular, we would just bring them up then I or any issues that we had then’ (Female*, 43 years, Parent, Usual Care, Site A*).*  *‘The check in meetings were quick and to the point, so it didn’t take long at all’ (Male, 24 years, Participant, ExACT, Site A).*  *‘The computer appointments were good, because then you could stay at home’ (Female, 11 years, Participant, ExACT, Site B).*  *‘I thought you guys were really flexible cause there was a time when I missed one of my afternoon video calls one Monday because I was travelling back from Liverpool – being able to reschedule it for later in the day wasn’t a problem’ (Male, 38 years, Participant, Usual Care, Site A).*  *‘We had the weekly, well mommy had the weekly calls, which was generally can you sync the watch please, so I can get the data? Yes, sorry. And you haven’t filled in all the days? No, I know, I’m doing it now. So, the weekly ones were probably quite useful for me, because it made me think, oh, I need to make sure everything is up to date. And they were just checking in, making sure everything’s okay’ (Female, 40 years, Parent, Usual Care, Site B).*  ‘*We’ve tried to be quite accommodating about times that they can be seen. So, we’ve done later visits and earlier visits, and calls at eight o’clock in the morning. And calls at six o'clock at night. Calls when they’re on holiday. So, we’ve tried to make it as easy for them as possible.’ (Female, 59 years, CTAP Nurse, Site B).*  *Challenges:*  *‘The Near Me could be glitchy’ (Female, 38 years, Parent, ExACT, Site A).*  *‘trying to pin them down, you know they’ve got different activities after school and they want to go out and play with their friends and all of a sudden they’ve got to come home for a call...So we tried to do a lot of the calls after school. Well, we had to do them after school, to try and fit in with their lifestyles. But then, that didn’t always work with our time schedules, so people were staying later. It was a little bit difficult...for the adults with people working, fitting in around their work meetings’ (Female, 41 years, Paediatric Research Nurse, Site B).*  *‘in most of my studies, we’ll have phone call plus or minus one or plus or minus two, just because it’s very hard to keep someone on the same day. Whereas your protocol doesn’t have anything and so that meant everything was a deviation. But in reality, if you were doing a larger scale, I don’t think you’d be able to write deviations for every single phone call that wasn’t done on a certain day’ (Female, 41 years, Paediatric Research Nurse, Site B).*  *‘for an awful lot of them doing it at the same time every week or even on the same day didn’t fit in with exams or various things. ..It’s supposed to represent your life, yes. And I think it did represent real life and sometimes we were tearing our hair out saying how are we going to fit this one in. So it represented real life from a staffing point of view as well, as to the coverage of staffing that we had.’ (Female, 55 years, Paediatric Research Nurse, Site B).* |
|  | Communication and relationship with the Study team | *‘I enjoyed working with the research team’ (Female, 14 years, Participant, Usual Care, Site A).*  *‘You quite liked the personalness of it, didn’t you? Like, when we first walked in and you seen the wee XXXXXX sign and things like that made her feel quite important to be part’ (Female*, 43 years, Parent, Usual Care, Site A*).*  *‘I think for me, it was all very streamlined, but then again I don’t know if that’s because I’ve been used to that, because we’ve been dealing with the team for so long.’ (Female, 40 years, Parent, Usual Care, Site B).*  *‘I thought the communication with the team was really good’ ( Female, 12 years, Participant, Usual Care, Site A).*  *‘They were always friendly, questions being answered really nicely, welcome on arrival... We knew who to contact if we had any concerns.’ (Female, 52 years, Parent, Usual Care, Site A).*  *‘I thought the research team were great. Everybody was really helpful.’ (Male, 38 years, Participant, Usual Care, Site A).*  *‘it was really good –I was able to contact anyone and everyone if I had any issues’ (Female, 23 years, Participant, ExACT, Site B).*  *Challenges:*  ‘*… they [other sites] were probably experiencing the same issues and difficulties as us, and we could have maybe supported and helped each other a bit more with how things are going. We’ve not really discussed how their recruiting, how are they getting on? What tips have they got for troubleshooting? Or we could have maybe shared ideas’ (Female, 59 years, CTAP Nurse, Site B).*  *‘The email address has been great for communication, but what would be really good would be to have a study phone’ (Female, 59 years, CTAP Nurse, Site B).* |

**Table S2.** Additional illustrative quotes from Theme 2: Towards choice and flexibility: evolving perspectives on airway clearance.

| **Subthemes** | | **Additional illustrative quotes** |
| --- | --- | --- |
| **Views around traditional Physiotherapy** |  | *‘I think I would always prefer exercise over the traditional physio just because it – it’s just not as prescribed is it? It feels like something you are choosing to do more than something you have to do. Plus I like the overall benefits of exercise as a lifestyle thing, not just a targeted CF thing’ (Male, 38 years, Participant, Usual Care, Site A).*  *‘I feel like you need to also actually like it because if you like doing it, it’s gonna be great, but if you don’t really like doing it you’re not going to try that hard and it’s like saying ‘I’m doing gymnastics but I hate gymnastics, so I’m not going to try hard enough’ but if I’m doing swimming and I love swimming, I’m going to try really, really hard on it. It’s like that with the Aerobika and the bike and the treadmill and it’s like if you don’t like it you’re not going to try that hard cause you’re like ‘I don’t like this. I’m just gonna do it quickly and get it over and done with.’ If you like it you’re gonna be like ‘Right, I’m ready, I’m gonna do this today. I’m doing this, and I’m gonna do it perfectly the first time’, and it’s you’ll be really really happy to do it’ (Female, 10 years, Participant, ExACT, Site A).*  *‘a lot of our patients with CF already do swap in some of their airway clearance times, if you like, the times they were meant to be doing (traditional) airway clearance for exercise’ (Female, 54 years, Physiotherapist, Site A).*  *‘..it would be nice for the physios to be able to devise a routine for them that includes exercise so that they’re not constantly being told they’re not adhering, because I think that can be quite damaging’ (Female, 29 years, Research Nurse, Site B).*  ‘*Some very purist physios maybe are not on board with that and would be very much ‘no, you still need to do your airway clearance as well’ but for years we’ve had patients who have asked about it’ (Female, 43 years, Paediatric Physiotherapist, Site A).*  When asked about a preference - *‘I would say exercise. Over the years, I’ve never really done any of the physio, and when I have tried to do it it’s not been very engaging. And I’ve stopped it since, I guess, I don’t find it as engaging or as impacting as exercising.’ (Male, 24 years, Participant, ExACT, Site A).* |
| **Views around exercise as an airway** **clearance technique (ExACT)** | General | *‘So I think, yes, I think encouraging exercise for everybody is really good, especially if they can do things as a family or those kinds of things. Or some of them were going out, dad was taking them out on a run and they were riding their bike and he was running or whatever. Yes, those kind of things are good for all aspects of wellbeing, really.’ (Female, 55 years, Paediatric Research Nurse, Site B).* |
|  | Convenient and easy to embed | *‘I was able to fit all the sessions in and it didn't disrupt my routine’ (Female, 10 years, Participant, ExACT, Site A).*  *‘I was able to fit in 20 minutes every day into my daily routine okay’ (Female, 16 years, Participant, ExACT, Site A).*  *‘It frees up my mornings and afternoons’ (Female, 11 years, Participant, ExACT, Site B).*  *‘20 minutes once a day was fine.. I do running and swimming anyway…it made me feel a lot better running every day’ (Female, 16 years, Participant, ExACT, Site A).* |
|  | Safe and effective | ‘*Yeah there were no issues at all like that. I wasn’t concerned or worried about it.’ (Male, 24 years, Participant, ExACT, site A).*  *‘I was comfortable with it... she’s actually physically fit, and she likes exercise. And at that point, I was happy for her to drop her normal physio, because she’s quite well. Because she’s well in herself, it was a good time to do it’ (Female, 38 years, Parent, ExACT, Site B).*  *‘I progressively found myself maybe less breathless or able to perform a bit harder or a bit longer than maybe the week before, so I would say that physically it became less taxing the longer into the trial.’(Male, 24 years, Participant, ExACT, Site A).*  *‘I felt really confident- yeah, I wasn't hesitant at all. I just kind of straight into it to be honest... Mainly because I relied on, exercise myself my whole life, and where I've been a swimmer and stuff. So yeah, so I've always known what exercise can do like, and how helpful it can be in terms of like airway clearance and managing CF.’ (Female, 23 years, Participant, ExACT, Site B).*  *‘I knew it was working …because I'm actually producing sputum….I'm not really a productive person but doing it as much as I can every day, or the majority of days of the week- I was bringing up sputum and mucus and shift in it. So that just showed that showed to me that’s working’ (Female, 23 years, Participant, ExACT, Site B).* |
|  | Variability of exercises, greater enjoyment and motivation | *‘we all choose an exercise that we can fit in to our own daily lives and you know, that might be completely different to you from me, and you know so I suppose exercise means different things to different people whether its – somebody might like a dance class, somebody else might like track and field sort of, you know stuff. It’s so vast, isn’t it? But I think there’s something out there for everybody that it’s just how you engage’ (Female, 49 years, Paediatric CF Nurse, Site A).*  *‘I knew that she’d be more motivated to do exercise, than to do her PEP boring physio’(Female, 38 years, Parent, ExACT, Site B).*  *‘I found it a lot more enjoyable- because obviously like it's not just the health aspect of exercises, the other things as well’. (Female, 23 years, Participant, ExACT, Site B).*  *‘The positive, for me, was, she was happy to do the exercise, it was less pressure on us to be on her to do her PEP, and have to do that, and keep going on at her.’ (Female, 38 years, Parent, ExACT, Site B).*  *‘I feel like you need to also actually like it because if you like doing it, it’s gonna be great, but if you don’t really like doing it you’re not going to try that hard … if you don’t like it you’re not going to try that hard cause you’re like ‘I don’t like this. I’m just gonna do it quickly and get it over and done with.’ If you like it you’re gonna be like ‘Right, I’m ready, I’m gonna do this today. I’m doing this, this, this, this, and I’m gonna do it perfectly the first time’, and it’s you’ll be really really happy to do it’ (Female, 10 years, Participant, ExACT, Site A).* |
|  | Less burdomsome for parents | *‘We didn’t shout at each other for the whole trial. There was none of this ‘will you just get this done? Will you get this done?’ There was no stress in the morning, that was completely taken away ……It was lovely. It was great to see her happy doing something that was making her fit and well and active.’ (Female, 38 years, Parent, Site A).* |
|  | Feeling ‘normal’ | *A comment I got from quite a few different teenagers particularly was that being on the Kaftrio, they felt normal again and they didn’t want to keep doing something (traditional ACT) that was to do with their disease…. they were really struggling to do their physio, because there was no motivation. The word they used was complacent, because they felt well, they felt normal… Whereas a lot of them already do sport through school or after-school clubs or friends. And so, personally, I can see the appeal, because they’re doing it almost without knowing they’re doing it. Or they’re doing it with friends, so it doesn’t feel like it’s such a chore.’ (Female, 29 years, Research Nurse, Site B).*  *’We all like to advocate it, even pre-modulators, it was always one of the positive things we could say to parents, you know, that exercise is really good and it was one of these normal things where your child aren’t going to be asked to sit out at P.E. because they’ve got CF’ (Female, 49 years, Paediatric CF Nurse, Site A).* |
|  | Cost implications / weather dependant. | *‘because we’re in February, March, it’s cold and it’s dark you couldn’t just go for a run outside in the morning for 20 minutes....Luckily enough we are very fortunate and have the treadmill…* *I think as you go into the summer months there’s a lot more varied choice and options’ (Female, 38 years, Parent, ExACT, Site A).* |
|  | Can be challenging at times to fit into day | *‘…during the week it was no problem…the hardest was on the weekend because, I was quite busy some weekends. So although it was only 20 minutes it was still a bit harder to find the time…I managed to find time 95% of the days’ (Male, 24 years, Participant, ExACT, Site A).*  *‘Yes, and no, for the majority of parts it was manageable…but then there were other days, obviously like if I was working all day, for example, and I like got home, and I was just too-too tired- like too tired…I just couldn’t quite manage it’ (Female, 23 years, Participant, ExACT, Site B).* |
|  | Limited during periods of illness | *‘There’s probably times when, again, if people feel they’re feeling a bit worse, then they might think, I’ll do the active cycle of breathing and suchlike. But conversely, because they might not be well enough to do that intense 20 minutes of exercise, if you see what I mean...I think there’s a case for both at differing times...(Male, 45 years, Participant, Usual Care, Site B).* |
| **Personal choice / an interchange model** |  | *‘I would rather use them both because it’s – if I was really, really sick and ill and I chose to do only the bike and the treadmill, I wouldn’t feel up to doing it, so then I would want to do the aerobica because then it’s like, it’s easier because I’m blowing into my tube and then I’m huffing to get my lungs clear and good …but then if I was feeling great that day and I was like I want to do the treadmill or something I could do it and because it’s like you would then have variety of different things’ (Female, 10 years, Participant, ExACT, Site A).*  *‘someone asked me the other day, how do you know if you’re doing your job well, and I think it’s when a person with CF tells you that they do this when they’re not well or that when they’re feeling better. You know, when it’s not 4x 10 breaths five times a day whatever… you know it’s when they can escalate or de-escalate, I think that was the best, so interchangeably’ (Female, 43 years, Physiotherapist (paediatric care), Site A).*  *During periods of illness:*  *‘There’s probably times when, again, if people feel they’re feeling a bit worse, then they might think, I’ll do the active cycle of breathing and suchlike. But conversely, because they might not be well enough to do that intense 20 minutes of exercise, if you see what I mean...I think there’s a case for both at differing times...(Male, 45 years, Participant, Usual Care, Site B).*  *‘And it would be nice for the physios to be able to devise a routine for them that includes exercise so that they’re not constantly being told they’re not adhering, because I think that can be quite damaging’ (Female, 29 years, Paediatric Research Nurse, Site B).*  *‘I probably would, in the first instance, recommend both… So for example, if there was a patient doing twice-a-day airway clearance, what I might say to them is... If you’re doing twice a day physio, and that’s your routine, why not, on the days that you exercise, drop it to once a day physio and see how you get on? Try that for a couple of weeks, see how it goes… Keep an eye on things like your lung function, your sputum volume, how you’re feeling. If that gets worse, go back to what you normally do. If it stays the same, then you can think about maybe dropping off a little bit more physio. So try to give them a stepwise process of swapping one in for the other, rather than just going all-out.’ (Femalea, 54 years, Physiotherapist (adult care), Site B).*  *‘I think I would always prefer exercise over the traditional physio just because it – it’s just not as prescribed is it? It feels like something you are choosing to do more than something you have to do. Plus I like the overall benefits of exercise as a lifestyle thing, not just a targeted CF thing... However, I don’t think that traditional airway clearance is something that I will ever not do. I feel like it will always be something that I can go to if I’m feeling run down, if I’m feeling more congested than normal, I know I can do that.’ (Male, 38 years, Participant, Usual Care, Site A).* |
| **Need for more evidence & Personalised and educated choices** |  | *‘I think it would depend on the trial and what was involved in it. As well as how often I would have to come to appointments and check-ups and things like that. It wouldn’t necessarily be just the length of the trial that would be a deciding factor. It would be the whole thing, how it fitted in etc.. And if it was something that I wouldn’t do normally then I would probably have a think about if it was going to take too much time or something I’m not really too bothered about … there’s a whole range of things rather than just the length or duration of the trial’ (Male, 24 years, Participant, ExACT, Site A).*  *‘So it’s having an idea that what’s equivocal to an airway clearance session, then we can prove this and we can identify that there’s no detriment to a person from a pulmonary exacerbation frequency or from a long function perspective, no detriment to them doing exercise rather than airway clearance. I think that’s really positive message for the individuals with CF, their parents, their carers, and us as physios that are trying to influence how they care for their chest’ (Female, 54 years, Physiotherapist (adult care, Site B).*  *‘Let’s be able to choose, fast. So we are not on the Aerobika forever, that would be lovely, thank you very much’ (Female, 38 years, Parent, ExACT, Site A).*  *‘I was really pro exercise and I have just always been really honest with patients and said I can’t give an evidence-based answer to using it instead of airway clearance. So, 100% I would love you to do it as it’s beneficial for x, y, and z, but in terms of if you can actually stop your airway clearance for your exercise, we don’t have the research… we need these trials to be able to then give people the information so they can make a more informed decision. Which, right now, we don’t have the evidence to say that’ (Female, 34 years, Physiotherapist, Site A).*  *‘I have seen some patients who just made that decision to use exercise as their airway clearance for the day. And it was something that we didn’t discourage, as long as they recognised that it’s just one aspect of airway clearance, and that actually doing huffs and coughs post exercise, would be very beneficial to them…I was really hoping that you’d get some good results out of this trial. And then we can start to give our patients the option of, if you are doing exercises on certain days, that was sufficient. And then airway clearance on the days when you’re not doing exercise. So, a nice balance of it. (Male, 43 years, Physiotherapist, Site B).*  *‘I think a lot of our pwCF already do swap in some of their airway clearance times… at the moment in the CFTR era is- we’re a bit worried about saying, well, you don’t need to do any physio, because we don’t know what’s ahead of us and we don’t want to create any negativity going forward. ..So we say, okay, it’s okay, don’t do any physio. But then five years down the line, their chest deteriorated because they haven’t done anything and we’ve given our permission, if you like, to do that.’ (Female, 54 years, Physiotherapist (adult care), Site B).* |
| **Future studies** |  | *‘I think it would depend on the trial and what was involved in it. As well as how often I would have to come to appointments and check-ups and things like that. It wouldn’t necessarily be just the length of the trial that would be a deciding factor. It would be the whole thing, how it fitted in etc.. And if it was something that I wouldn’t do normally, then I would probably have a think about if it was going to take too much time or something I’m not really too bothered about … there’s a whole range of things rather than just the length or duration of the trial’ (Male, 24 years, Participant, ExACT, Site A).* |

**S2.** Semi-structured interview guides used within the ExACT-CF study.

**ExACT-CF Interview Schedule**

**For participants and families**

**Date: …/…/…. Participant ID:**

**Introduction:**

- Re-introduce self and purpose of interview.
- Check consent with participant
- Remind the participant
  - Their responses will be kept confidential and that any direct quotes will not be used to identify them as an individual.
  - They can change their mind about taking part in the study, can stop the interview at any time or decline to answer a question without giving a reason.
  - Remind them that the interview will take approximately 15-20 minutes.
- Give the participant time to ask questions if they have any.

**TURN ON THE RECORDER**

- **Experience of Recruitment / Consent Process:**

1. Can you tell me about your experiences of joining (being recruited to) the trial:
   1. Who asked you to join, how and where?
   2. What did you think of the information leaflet you were given, did it clearly explain the study? Was there anything else you would have liked to know or would you add anything else to it?
2. Why did you decided to take part in the trial?

- **Experience of Randomisation Process and Assignment to Arm 1 or 2:**

1. Did you have a strong opinion or preference for exercise or traditional airway clearance before the trial and if so which?
2. What were your first thoughts about the group you were put into?
3. What did you think about the information you were given about your group in the trial:
   1. Did you receive enough information about what you should do on a daily basis?
   2. Would there have been any other information you feel would have been helpful?
   3. Did you know what to do or who to contact if you were concerned or had any questions?

- **Experience of intervention ExACT:**

1. What did you think about the different exercises you could do?
   1. Did you prefer any type of exercise?
   2. Was there any other exercise that you would usually do that was not included in the list?
2. Did you have any difficulties in using exercise as a replacement for traditional airway clearance techniques:
   1. Were you able to fit the exercise sessions within your usual daily routine or did you have to change anything, did you miss any sessions and if so why?
   2. Was the number of sessions appropriate or would you make any changes?
   3. Were there any barriers such as cost implications or access to equipment?
3. How confident and safe did you feel replacing traditional airway clearance with exercise?
4. How do you know (or think you would know) whether exercise is working or not for airway clearance?
5. Did you notice any changes to your sputum production during or following your exercise session(s)?
6. Did you see any other effects either positive or negative about exercising?

- **Experience of daily outcome measures.**

1. How did you find the daily recording of your activities;
   1. Garmin watch – uploads
   2. Daily diary of exercise type and frequency.
2. Do you have any suggestions as to how to record this activity in an easier way?
3. What do you think are the most important things to measure in order to tell whether exercise is working or not for airway clearance?

- **Experience of other assessments and questionnaires.**

1. What do you think of the assessments and questionnaires that you had to complete:
   1. Did you think they were all relevant?
   2. What did you think of the format (paper / digital)
   3. Were you given enough time and support to complete them?
2. Which assessment or questionnaire did you feel was most important and why?

- **Overall experience of participating in the trial.**

1. Going forward would you rather do exercise / traditional airway clearance / or use them both interchangeably and why?
2. How was your overall experience within the trial:
   1. working with the research team in terms of communication and support?
   2. Ease of taking part such as time, effort, adherence and commitment?
   3. What did you enjoy the most?
   4. What parts did you find the hardest?
3. Did you drop out of the trial, if so why?
4. If a longer trial was to take place what suggestions would give to make it easier or more enjoyable for people?
5. Would you be happy to take part in a randomised trial again?
   1. If so would you be happier to take part in a longer randomised trial, for 6 months or 1 year?

End:

- Thank the participant for taking part
- Explain what will happen next with their data and right to withdraw
- Allow time for any questions

**ExACT-CF Interview Schedule**

**For health professionals recruiting to and supporting participants in ExACT-CF**

**Date: …/…/…. Participant ID:**

**Introduction:**

- Re-introduce self and purpose of interview.
- Check consent with interviewee
- Remind the interviewee:
  - Their responses will be kept confidential and that any direct quotes will not be used to identify them as an individual.
  - They can change their mind about taking part in the interview at any time or decline to answer a question without giving a reason.
  - Remind them that the interview will take approximately 15-20 minutes.
- Give the interviewee time to ask questions if they have any.

**TURN ON THE RECORDER**

- **Experience around Identification, Recruitment and Consent.**

1. Please tell me about your experience of identifying and trying to recruit patients to the ExACT-CF study:
   1. How did you find the recruitment leaflet – e.g. was everything clear, was there any information missing which you though would have been beneficial to include?
   2. What did you find were the main challenges to recruiting participants?
   3. Do you have any suggestions for improving the recruitment process in order to encourage more patients to take part?

- **Experience of Randomisation Process and assignment to Arm 1 or 2.**

1. Were you involved in taking patients through the randomisation process, if so
   1. What was your experience of dealing with participants who were about to be randomised, were you aware of any concerns?
   2. Did patients understand it, were there any questions?
2. Did you feel you were given enough information to advise the participants / understand what they should do on a daily basis?
3. Would there have been any other information you feel would have been helpful?

- **Experience of Intervention ExACT.**

1. Before the trial what were your thoughts about using exercise as airway clearance?
2. How do you think pwCF (participants) felt about exercise replacing chest physiotherapy?
3. What were your expectations of ExACT intervention.
   1. Was the intervention what you thought it was going to be?
   2. Were there any concerns raised?
   3. Did you receive any feedback from your patients?

- **Experience of other assessments and questionnaires.**

1. What did you think of the assessments and questionnaires that the participants needed to complete.
   1. Did you think they were all relevant?
   2. What difficulties do you think the participants experienced in completing the questionnaires or activity logs such as time?
   3. What did you think of the format (paper / digital)??
2. Which assessment, activity log or questionnaire did you feel was most important and why?
3. Did you think the outcome measures captured participants experiences or was there anything missing? Could you suggest any additional beneficial outcome measures?
4. What are your suggestions for improving questionnaires, so that all participants can complete them easily? E.g. (paper/digital).

- **Experience of the study database.**

1. How easy is / was it to use?
   1. Registering new participants
   2. Entering study visit data
2. Could you see this database being transferable to a larger scale trial?
3. For nurses not familiar with the study, do you think that they could use the database?
4. How does the database compare with those that you have used on other trials?
5. Do you have any suggestions to improve the data base for future use in further studies?

- **Overall experience of the trial.**

1. Going forward how do you feel about recommending exercise instead of traditional chest physiotherapy for airway clearance?
2. What do you feel would be optimal treatment exercise or traditional airway clearance or using exercise and airway clearance interchangeably?
3. How was your overall experience working with the research team in terms of communication and support?
4. What do you feel are the main reasons people declined to participate / dropped out or withdrew from the study?
5. If a longer trial was to take place what suggestions would you make to so it was easier or more enjoyable for people / participants?

End:

- Thank them for their time.
- Explain what will happen next with their data and right to withdraw
- Allow time for any questions

**ExACT-CF Interview Schedule**

**For individuals who were screened but did not give their consent to be included in ExACT-CF**

**Date: …/…/…. Participant ID:**

**Introduction:**

- Re-introduce self and purpose of interview
- Check consent with interviewee
- Remind the interviewee
  - Their responses will be kept confidential and that any direct quotes will not be used to identify them as an individual.
  - They can stop the interview at any time or decline to answer a question without giving a reason.
  - Remind them that the interview will take approximately 5-10 minutes.
- Give the interviewee time to ask questions if they have any.

**TURN ON THE RECORDER**

1. How was your experience of being approached to take part in the ExACT CF study and why did you decide not to take part?
2. If there was a similar future trial, do you have any suggestions in order to encourage more people to take part in it?
3. If you had been able to choose which group (arm) of the study you would be in, would that have changed your mind about participating in the study?
4. How do you generally feel about exercise and physical activity?
5. What are your thoughts about using exercise instead of traditional airway clearance? Do you prefer exercise, traditional airway clearance or would you rather use them interchangeably?

End:

- Thank the interviewee
- Explain what will happen next with their data and right to withdraw
- Allow time for any questions
